# High-Entropy Pyrochlore A_2_B_2_O_7_ with Both Heavy and Light Rare-Earth Elements at the A Site

**DOI:** 10.3390/ma15010129

**Published:** 2021-12-24

**Authors:** Zhen Teng, Yongqiang Tan, Haibin Zhang

**Affiliations:** 1 Department of Materials Science, Fudan University, 220 Handan Road, Shanghai 200433, China; tengzhen426@163.com; 2 Innovation Research Team for Advanced Ceramics, Institute of Nuclear Physics and Chemistry, China Academy of Engineering Physics, Mianyang 621900, China; tanyongqiang1986@163.com

**Keywords:** high-entropy ceramics, pyrochlore, zirconate, configuration entropy

## Abstract

A novel class of high-entropy pyrochlore ceramics (HEPCs) with multiple heavy and light rare-earth elements at the A site were successfully synthesized via solid-state reaction. Both the XRD patterns and Raman spectroscopy demonstrated the single pyrochlore structure feature of seven kinds of HEPCs. Electron microscopic images revealed the typical morphology and the homogeneous distribution of all rare-earth elements. It can be concluded that the significance of configuration entropy in the HEPC system has promoted the tervalent lanthanide nuclides to form a single pyrochlore structure. This work is expected to provide guidance for the further design of high-entropy pyrochlore/fluorite ceramics.

## 1. Introduction

Since Rost and co-workers took the lead in synthesizing entropy-stabilized oxides with rock-salt phase (Mg_0.2_Zn_0.2_Cu_0.2_Co_0.2_Ni_0.2_)O in 2015 [1], the concept of high-entropy materials was extended from alloys to non-metallic materials, known as high-entropy ceramics (HECs). HECs including diborides, carbides, nitrides and oxides have received considerable attention [2,3,4,5]. Great efforts have been devoted to preparing HECs, owing to their superior structural or functional properties which result from severe lattice distortion and high compositional complexity, just like high-entropy alloys [6,7,8]. Among them, high-entropy pyrochlore/fluorite ceramics have attracted ever-increasing interest because of their lower thermal conductivity, higher chemical durability and better thermal stability [9,10,11,12].

The general formula of the pyrochlore crystal structure is A_2_B_2_O_7_ (Space group Fd$\bar{3}$m), superstructures of the fluorite structure (Fm$\bar{3}$m), where A can be trivalent lanthanides and B can be tetravalent Ti, Zr, and Hf. The A and B site cations in eight- and six-fold coordination form a face-centered cubic array and are ordered on two distinct cation sites into rows in the <110> direction. Due to their structural flexibility [13,14], hundreds of HEPCs have been synthesized in the past few years and their family is still expanding [15,16]. For example, some researchers discovered unique properties for the HEPCs, such as significantly reduced thermal conductivity, improved thermal expansion coefficient, and excellent chemical durability, due to their complex structure and severe lattice distortion. In addition, some have put forward the rules of pyrochlore–fluorite phase transition [17,18,19,20]. Nevertheless, previous reports mainly focused on the evolution of performance with several heavy or light rare-earth elements [21,22]. For the HECs, on the one hand, due to a large amount of configuration entropy, the Gibbs free energy of the system can be reduced and the stability of the system is improved. On the other hand, the large lattice distortion leads to the segregation of elements with large differences in ionic radius, therefore, it is difficult to obtain a single-phase structure [23,24]. Generally, similar chemical characteristics, close ionic radii, as well as the average radius ratio r_A_/r_B_ rule need to be considered to obtain a single high-entropy pyrochlore structure [25]. Therefore, the systematic research on the influence of structure with doped lanthanide nuclides, including heavy and light rare-earth elements, may provide valuable guidance for the design of high-entropy pyrochlore/fluorite ceramics.

Herein, several kinds of HEPCs composed of light and/or heavy rare-earth elements were synthesized. The (La_1/5_Nd_1/5_Sm_1/5_Eu_1/5_Gd_1/5_)_2_Zr_2_O_7_, which owns the largest average ion radius of the A site and is composed of light rare-earth elements, was synthesized first. Considering the valence and ionic radius, tervalent heavy rare-earth elements (Dy, Ho, Er, Tm, Yb, Lu) were applied to replace the La element to study the influence of elements with a larger ion radii difference on the crystal structure. The XRD patterns and Raman spectra were collected to detect and confirm the crystal structure of HEPCs. SEM, with the corresponding EDS mapping, was applied to observe the distribution of the rare-earth elements. HR-TEM was used to study the detailed microstructure of the HEPCs.

## 2. Experimental Procedure

### 2.1. Synthesis of High Entropy Pyrochlore Oxides

Commercial La_2_O_3_, Nd_2_O_3_, Sm_2_O_3_, Eu_2_O_3_, Gd_2_O_3_, Dy_2_O_3_, Ho_2_O_3,_ Er_2_O_3_, Tm_2_O_3_, Yb_2_O_3_, Lu_2_O_3,_ and ZrO_2_ (Aladdin, 99.99% purity) powders were employed as the raw materials. Multicomponent HEPCs were synthesized via a solid-state reaction method. After accurate weighing, ball milling, and drying, the mixed powders were compressed in a ¾− inch-diameter stainless mold and cold isostatic pressed at 200 MPa for 5 min. Finally, the green samples were sintered at 1500 °C for 40 h in air and cooled to room temperature, which is similar to the procedure reported in our previous work [26].

### 2.2. Characterization

X-ray diffraction (XRD, Bruker D8 Discover) with Cu Kα radiation (*λ* = 1.54 Å) was used to identify the crystal structure and phase composition of the oxide sample with a step scan of 0.02° and 10 s per step. XRD refinement was performed by Rietveld refinement software (Fullprof). A He-Ne laser (*λ* = 532 nm) was applied to record Raman spectra on a confocal Raman spectroscopy system (Renishaw, Wotton-under-Edge, UK, RM-1000) in the range of 200–800 cm^−1^. The elemental distribution of the sample was performed on a field emission scanning electron microscope (SEM, Zeiss Supra 50VP, Germany) with an energy dispersive X-ray spectrometer (Oxford EDS, with INCA software). A transmission electron microscope (TEM, JMF-2100F) was used to analyze the morphology of HEPCs with an accelerating voltage of 200 keV.

## 3. Results and Discussion

**Figure 1:**
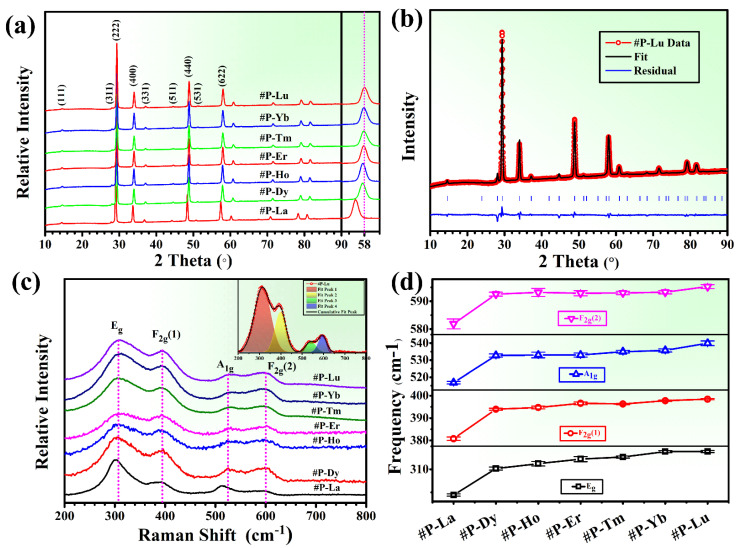
(**a**) XRD patterns of compounds #P-La~Lu. (**b**) XRD patterns together with Rietveld fit of #P-Lu. (**c**) Raman spectra of compounds #P-La~Lu. The inset shows the Gaussian peak fit of #P-Lu. (**d**) Raman shift with corresponding error bars of four Raman modes of compounds #P-La~Lu.

For the sake of facilitating presentation, as shown in Table 1, we named them: #P-La, #P-Dy, #P-Ho, #P-Er, #P-Tm, #P-Yb, #P-Lu, where P refers to pyrochlore and the latter elements refer to newly incorporated elements. The XRD patterns of the seven HEPCs #P-La~Lu are presented in Figure 1a. It can be seen that these seven compounds exhibit a single pyrochlore structure without any secondary phases, which could be demonstrated by typical super-lattice diffractions of 2 θ at approximately 15°, 29°, 37°, 44° and 51° [25]. As we all know, the ratio of the average ionic radius of A and B cations (r_A_/r_B_) is one of the critical factors for high-entropy fluorite-pyrochlore transition [27]. When the r_A_/r_B_ value extends from 1.46 to 1.78, the pyrochlore structure becomes stable. When the average radius ratio r_A_/r_B_ is less than 1.46, it would transform into a defective fluorite structure. The radii of La^3+^, Nd^3+^, Sm^3+^, Eu^3+^, Gd^3+^, Dy^3+^, Ho^3+^, Er^3+^, Tm^3+^, Yb^3+^, and Lu^3+^ in eight-fold coordination are 1.16, 1.109, 1.079, 1.066, 1.053, 1.027, 1.015, 1.004, 0.994, 0.985 and 0.980 Å, respectively [28]. As shown in Table 2, the r_A_/r_B_ values of the seven compounds lay in the range from 1.4686 to 1.5158, which is consistent with the previous discussion. Meanwhile, as shown in the enlarged part of Figure 1a, the diffractions peaks shifted to the right from #P-La to #P-Lu. According to Bragg’s Law [29], when an element with a smaller ionic radius is successfully dissolved in the crystal lattice, the lattice constant would become smaller, leading to the right-shift of diffraction peaks. The radii of Er^3+^, Tm^3+^, Yb^3+^, and Lu^3+^ in eight-fold coordination are 1.004, 0.994, 0.985 and 0.980 Å, which are very close and smaller than La^3+^ (1.16 Å). Thus, the shift of diffraction peaks for the #P-Er~#P-Lu were not obvious, and this can also be seen in the refinement of lattice parameters in Table 2. Therefore, the XRD results confirmed to a certain extent that all the rare-earth elements had successfully entered into the A sites.

Figure 1b shows the XRD patterns together with the Rietveld fit of #P-Lu. As can be seen, the refined curve of #P-Lu matched quite well with the XRD curve, indicating that the refined results are reliable. The XRD results of the seven HEPCs were refined using the Rietveld method, and the main refined parameters are listed in Table 2. The lattice parameters decreased from 10.64364 to 10.54532 Å, which was similar to the previous report [24].

Raman spectroscopy is an effective tool to investigate the vibration and rotation information of chemical bonds in the pyrochlore crystal structure. Figure 1c displays the Raman spectra of the seven compounds. In the range of 200–800 cm^−1^, the seven compounds exhibit four typical Raman vibration modes at 315, 400, 525, and 590 cm^−1^, respectively, corresponding to four vibration modes $E_{g}$, $F_{2g}\left(1\right)$, $A_{1g}$, and $F_{2g}\left(2\right)$ [30]. The Raman vibration modes also confirm the seven compounds are pyrochlore structures from the perspective of chemical bonds.

Due to the overlap of the four Raman modes, the Gaussian fit was used to analyze the detailed shift of the four Raman vibration modes. Figure 1c shows the four Raman modes together with the Gaussian fit of #P-Lu and the cumulative fit peaks fitted well with the experimental line. Figure 1d displays the Raman shift with corresponding error bars of the four Raman modes for the compounds #P-La~Lu. It can be observed that the four Raman modes move to a higher frequency (blue shift) from #P-La to #P-Lu, which indicates the shortened bond length and the strengthening of the corresponding chemical bond [31]. It is worth noting that the shift of the Raman modes and the shift of the X-ray diffractions peaks show a very similar tendency, suggesting that the blue shift of the Raman modes is mainly caused by the decrease in lattice parameters.

**Figure 2:**
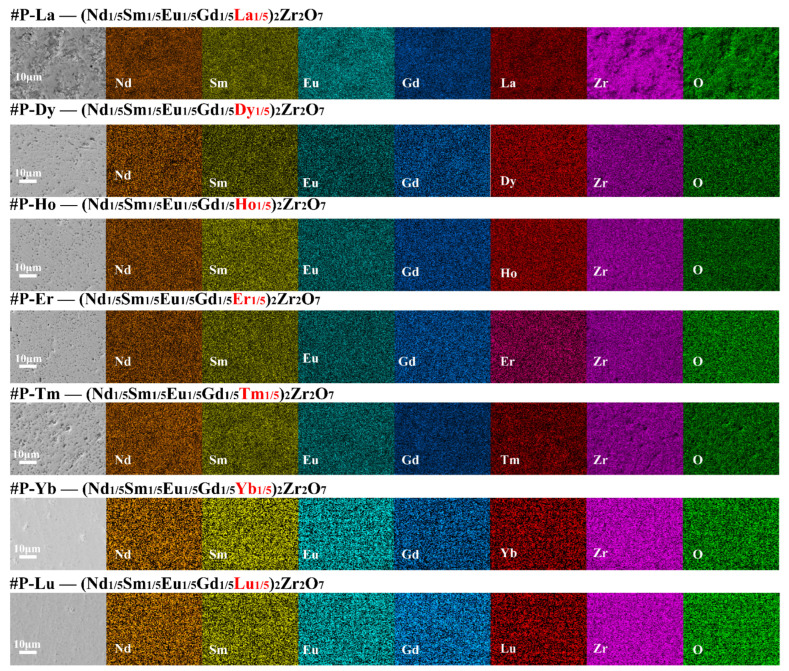
Cross-section SEM images and the corresponding EDS mapping of the seven compounds.

Figure 2 displays cross-section SEM images and the corresponding EDS mapping of the seven compounds. It can be observed that the elements of La^3+^, Nd^3+^, Sm^3+^, Eu^3+^, Gd^3+^, Dy^3+^, Ho^3+^, Er^3+^, Tm^3+^, Yb^3+^, Lu^3+^, Zr^4+^, and O^2+^ are all distributed randomly and homogeneously, which indicated the good composition uniformity of the seven compounds. Furthermore, the significance of porosity can be seen on the surface of the compounds, which indicated low density, especially #P-La and #P-Tm. On the one hand, due to the strong covalent bond and low diffusion coefficient in the oxides, it usually only relies on solid-phase mass transfer to achieve densification. On the other hand, it is probably due to the large lattice distortion in the high-entropy system that further hinders the solid-phase mass transfer rate.

**Figure 3:**
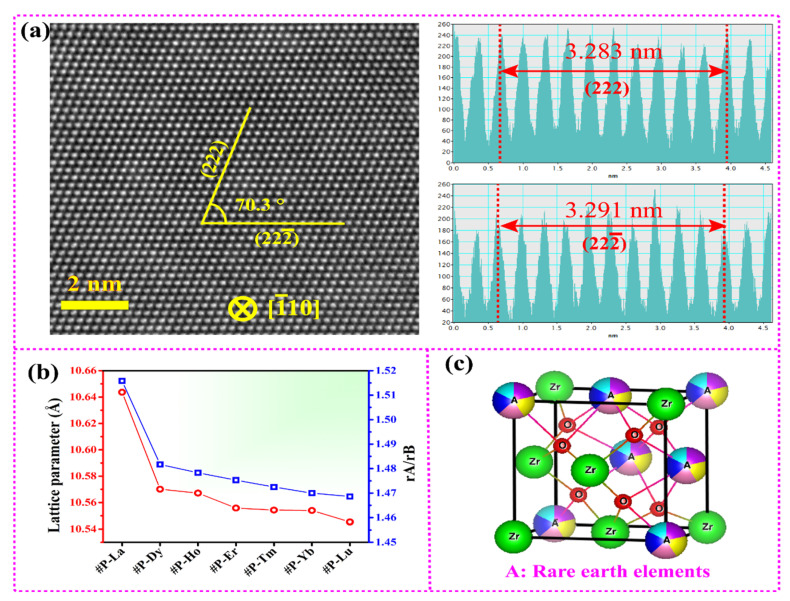
(**a**) HR-TEM images of #P-La. (**b**) The ionic radius ratio and the lattice parameter of compounds #P-La~Lu. (**c**) The schematic diagram of the ideal high-entropy pyrochlore structure using VESTA.

Figure 3a shows the atomic-resolution transmission electron microscopy image of #P-La. It can be seen that the image exhibits sharp lattice fringes along the [110] zone axis, which reveal excellent pyrochlore lattice integrality. Meanwhile, the lattice parameter measured by the HR-TEM image was 10.645 Å, which is consistent with the refined results in Table 2. In addition, Figure 3b displays the correlation between the ionic radius ratio and the lattice parameter of the seven compounds. It can be seen that the two curves show a consistent trend and the results further show the successful doping of all the rare-earth elements.

By comprehensive consideration of the XRD patterns, Raman spectroscopy, SEM images and HR-TEM images, the schematic structure of the high-entropy pyrochlore is presented in Figure 3c. It can be seen that the Zr element occupies the location of 16d (1/2, 1/2, 1/2). Five rare-earth elements equally and homogeneously occupy 16c (0, 0, 0), and the oxygen occupies 8b (3/8, 3/8, 3/8) and 48f (x, 1/8, 1/8). In addition, there is an oxygen vacancy occupying the location of 8a (1/8, 1/8 1/8). Thus, the location of O_48f_ plays a decisive role in the crystal structure of pyrochlore. Generally, the x is located between 0.3125 (ideal pyrochlore) and 0.375 (defect fluorite) [25]. In Table 2, the x-parameters of the seven oxides were around 0.35, which further clarified that the seven oxides are highly ordered pyrochlore structures.

The lanthanides in this work show similar chemical characteristics, close ionic radii and the same valence, which were considered candidates for providing HEPCs. Thus, the atomic size difference (*δ*) was used to represent the difference in atomic radii, which was first proposed by Zhang et al. and used in the high-entropy alloys community [32]. The parameter δ can be defined as [27]:

$$\delta r=\sqrt{\sum^{\;}c_{i}{\left(1-r_{i}/\bar{r}\right)}^{2}}$$

where *r_i_* and *c_i_* are the atomic radii and atom fraction of elements *i*; $\bar{r}$ is the average atomic radius. Due to the lanthanides occupying the A sites, the *r_i_* and $\bar{r}$ represent the atomic radii and the average atomic radius of the A sites. As shown in Table 2, the atomic size disorder (*δ*) decreased from 3.18 to 2.55% and then increased from 2.55 to 4.06%. The crystal structure of the seven compounds remained a pyrochlore structure, which is consistent with previous reports. Just like high-entropy alloys [32], the atomic size disorder (*δ*) may have an upper limit for HEPCs (larger than 4.06%). Besides, considering previous reports [27], it can be speculated that any five tervalent light and/or heavy rare-earth elements can form a single pyrochlore structure with zirconium as the ion radius ratio r_A_/r_B_ ranges from 1.46 to 1.78. This is because the significance of configuration entropy in the high-entropy pyrochlore system has improved the stability of the system.

## 4. Conclusions

In this work, (1) a series of HEPCs with heavy and light rare-earth elements containing all tervalent lanthanide nuclides were successfully synthesized, which greatly expanded the family of the HEPCs. (2) The XRD patterns and Raman spectroscopy revealed that all the compounds had a single pyrochlore structure from the crystallography and chemical bonds. (3) The SEM image with the corresponding EDS mapping indicated that all the rare-earth elements were randomly and homogeneously distributed within the matrix. The HR-TEM image clearly displayed the typical morphology and complete microstructure of the high-entropy pyrochlore structure. (4) The significance of configuration entropy in the high-entropy pyrochlore system allows any five lanthanides to form a single pyrochlore structure with zirconium. This work is expected to guide the composition design of high-entropy pyrochlore/fluorite ceramics with lower thermal conductivity, in the future, such as lower thermal conductivity for thermal barrier coating, higher irradiation resistance, and chemical durability as potential waste forms for actinide immobilization.

## Figures and Tables

**Table 1:** Composition and corresponding abbreviation of the seven high-entropy pyrochlore oxides.

Abbreviation	Composition
#P-La	(Nd_1/5_Sm_1/5_Eu_1/5_Gd_1/5_La_1/5_)_2_Zr_2_O_7_
#P-Dy	(Nd_1/5_Sm_1/5_Eu_1/5_Gd_1/5_Dy_1/5_)_2_Zr_2_O_7_
#P-Ho	(Nd_1/5_Sm_1/5_Eu_1/5_Gd_1/5_Ho_1/5_)_2_Zr_2_O_7_
#P-Er	(Nd_1/5_Sm_1/5_Eu_1/5_Gd_1/5_Er_1/5_)_2_Zr_2_O_7_
#P-Tm	(Nd_1/5_Sm_1/5_Eu_1/5_Gd_1/5_Tm_1/5_)_2_Zr_2_O_7_
#P-Yb	(Nd_1/5_Sm_1/5_Eu_1/5_Gd_1/5_Yb_1/5_)_2_Zr_2_O_7_
#P-Lu	(Nd_1/5_Sm_1/5_Eu_1/5_Gd_1/5_Lu_1/5_)_2_Zr_2_O_7_

**Table 2:** Main parameters of the seven oxides.

Abbreviation	Lattice Parameter (Å)	*x*-Parameter	r_A_/r_B_	δr
#P-La	10.64364(26)	0.34416(79)	1.5158	3.18%
#P-Dy	10.57011(32)	0.35109(93)	1.4817	2.55%
#P-Ho	10.55579(30)	0.35412(95)	1.4783	2.90%
#P-Er	10.56713(28)	0.35504(94)	1.4753	3.25%
#P-Tm	10.55427(35)	0.35599(99)	1.4725	3.58%
#P-Yb	10.55395(32)	0.35444(92)	1.4700	3.89%
#P-Lu	10.54532(30)	0.35170(79)	1.4686	4.06%

*x*-parameter: the location of O_48*f*_ in the crystal structure of pyrochlore. r_A_/r_B:_ the ratio of the average ionic radius of A and B cations. $\delta_{r}$: the atomic size difference.

## Data Availability

The data supporting the conclusions of this article are included within the article.
